# Supplementary figures and images for: Exploring solid-phase proximity ligation assay for survivin detection in urine
Source: PLoS One. 2022 Jun 29;17(6):e0270535. doi: 10.1371/journal.pone.0270535 (PMC9242480; doi:10.1371/journal.pone.0270535)

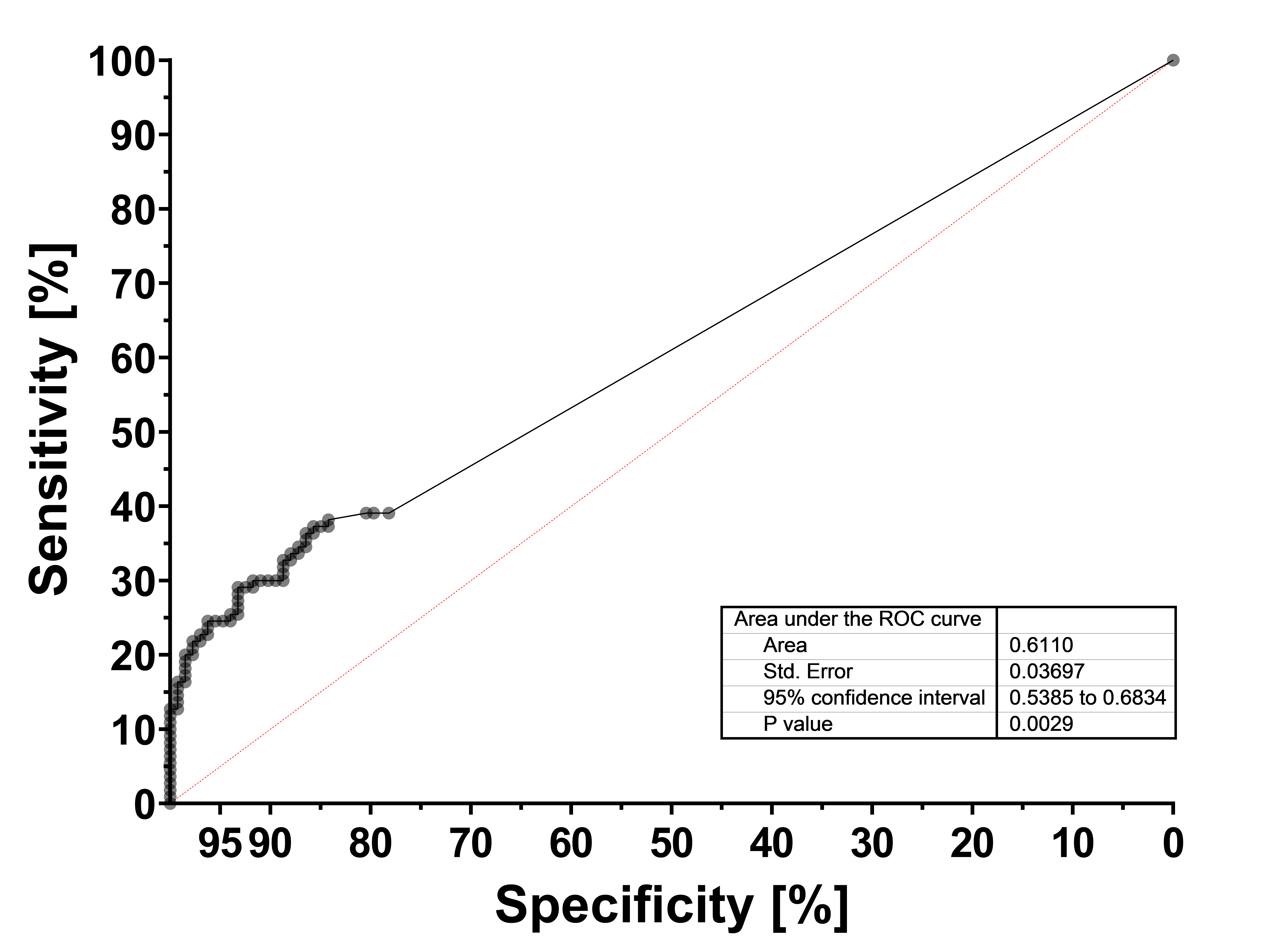

Supplement: S1 Fig — (TIF) [file pone.0270535.s001.tif]

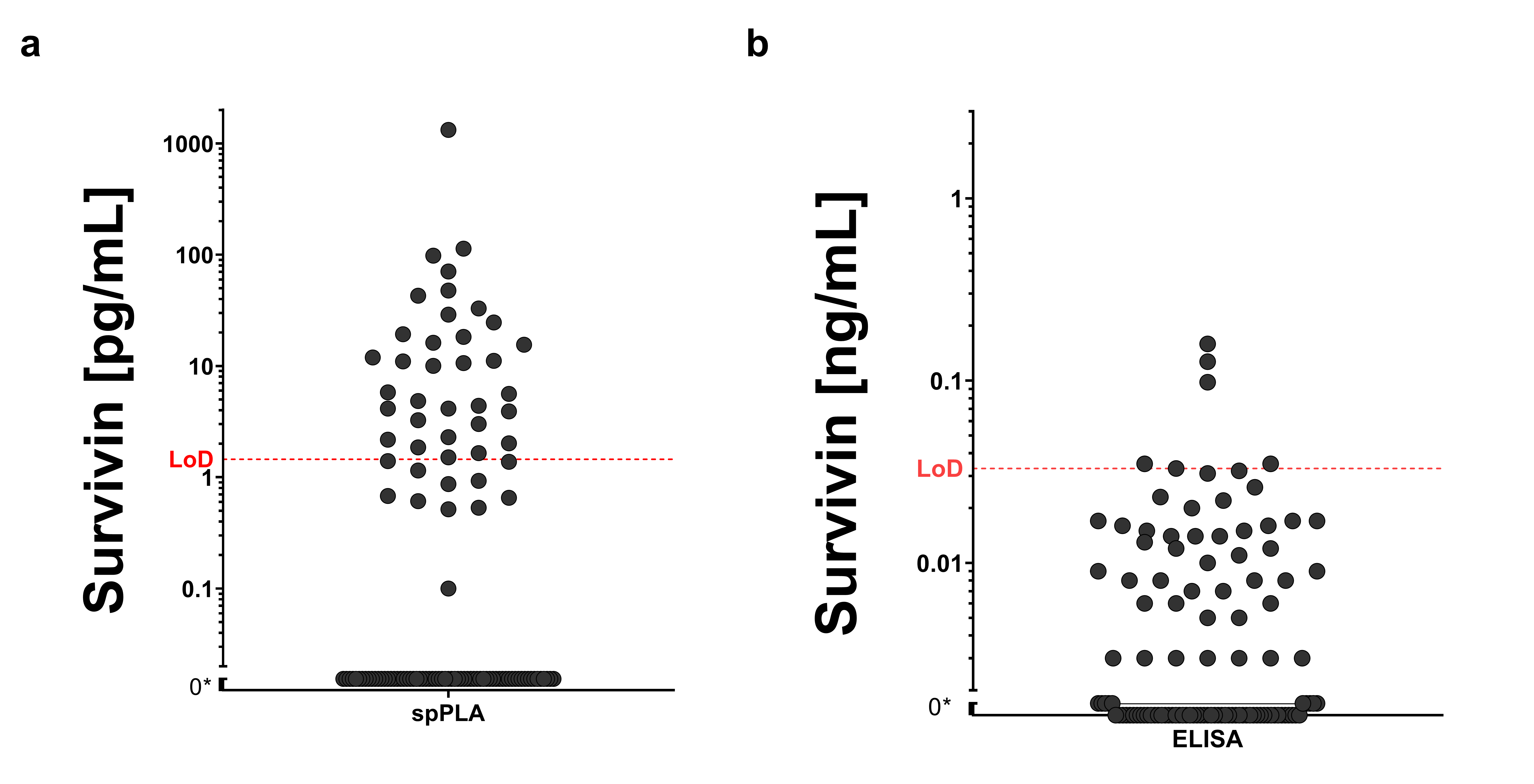

Supplement: S2 Fig — Detection of survivin in 500 μL voided urine from bladder cancer patients measuered by (a) spPLA and (b) ELISA. Samples containing no measurable amounts of survivin are indicated with 0*. LoD: Limit of Detection. (TIF) [file pone.0270535.s002.tif]
