# Supplementary material for: Exploring solid-phase proximity ligation assay for survivin detection in urine
Source: PLoS One. 2022 Jun 29;17(6):e0270535. doi: 10.1371/journal.pone.0270535 (PMC9242480; doi:10.1371/journal.pone.0270535)
Supplement: S1 Table — (DOCX) [file pone.0270535.s003.docx]

**S1 Table. Characteristics of the study group**

| Characteristics | All  (n = 243) | | | | Cases  (n = 110) | | | | Clinical Controls  (n = 133) | | | |
| --- | --- | --- | --- | --- | --- | --- | --- | --- | --- | --- | --- | --- |
| Sex | Male | | Female | | Male | | Female | | Male | | Female | |
| n | 174 | | 69 | | 82 | | 28 | | 92 | | 41 | |
|  |  |  | |  |  |  | |  |  |  | |  |
|  | n | Median | | IQR | n | Median | | IQR | n | Median | | IQR |
| Age (years) | 243 | 73 | | 63–80 | 110 | 74 | | 65–80 | 133 | 71 | | 60–79 |
| Body mass index | 243 | 26.4 | | 24.0–29.7 | 110 | 26.9 | | 24.3–30.4 | 133 | 26.3 | | 23.6–29.6 |
| Urine volume (mL) | 243 | 30 | | 20–50 | 110 | 20 | | 14–40 | 133 | 35 | | 20–50 |
| Urine retention within bladder (h) | 235 | 2.0 | | 1.0–3.0 | 105 | 1.75 | | 1.0–2.5 | 130 | 2.0 | | 1.0–3.5 |
| Specific gravity (g/L) | 239 | 1015 | | 1015-1020 | 108 | 1015 | | 1015–1020 | 131 | 1020 | | 1015–1020 |
| pH | 239 | 5 | | 5.0–6.5 | 108 | 5 | | 5–6.5 | 131 | 5 | | 5–6.5 |

IQR = interquartile range
