# Supplementary material for: Exploring solid-phase proximity ligation assay for survivin detection in urine
Source: PLoS One. 2022 Jun 29;17(6):e0270535. doi: 10.1371/journal.pone.0270535 (PMC9242480; doi:10.1371/journal.pone.0270535)
Supplement: S2 Table — (DOCX) [file pone.0270535.s004.docx]

**S2 Table. Characteristics of the 110 bladder cancer cases.**

| Characteristic | Status | n |
| --- | --- | --- |
| Tumor Stage | Ta | 60 |
|  | T1 | 14 |
|  | T2 | 23 |
|  | T3 | 9 |
|  | Carcinoma *in situ* | 3 |
|  | Missing | 1 |
| Histological grade | Low | 54 |
|  | High | 55 |
|  | Missing | 1 |
| Recurrent | Yes | 52 |
|  | No | 57 |
|  | Missing | 1 |
| Gross hematuria | Yes | 65 |
|  | No | 45 |
| Dysuria | Yes | 33 |
|  | No | 77 |
| Frequent urination | Yes | 51 |
|  | No | 59 |
| Bladder Stones | Yes | 12 |
|  | No | 98 |
| Diabetes mellitus type II | Yes | 12 |
|  | No | 98 |
| Smoking | Never | 23 |
|  | Former | 50 |
|  | Actual | 35 |
|  | Missing | 2 |
